# Transcranial Magnetic Stimulation as a Therapeutic Option for Neurologic and Psychiatric Illnesses

**DOI:** 10.7759/cureus.3456

**Published:** 2018-10-16

**Authors:** Sara Habib, Umair Hamid, Ayesha Jamil, Aariz Z Zainab, Tooba Yousuf, Sana Habib, Syed Maaz Tariq, Faryal Ali

**Affiliations:** 1 Neurology, Thomas Jefferson University, Philadelphia, USA; 2 Internal Medicine, The Indus Hospital, Lahore, PAK; 3 Internal Medicine, Fatima Jinnah Medical University, Lahore, PAK; 4 Radiology, Mayo Hospital King Edward Medical College, Lahore, PAK; 5 Family Medicine, Civil Hospital Karachi, Dow University of Health Sciences, Karachi, PAK; 6 Internal Medicine, Dow University of Health Sciences, Karachi, PAK; 7 Internal Medicine, Jinnah Sindh Medical University, Karachi, PAK

**Keywords:** electromagnetic, transcranial, stimulation, electrophysiology

## Abstract

In recent years, transcranial magnetic stimulation has become an area of interest in the field of neurosciences due to its ability to non-invasively induce sufficient electric current to depolarize superficial axons and networks in the cortex and can be used to explore brain functioning. Evidence shows that transcranial magnetic stimulation could be used as a diagnostic and therapeutic tool for various neurological and psychiatric illnesses. The aim of this review is to introduce the basics of this technology to the readers and to bring together an overview of some of its clinical applications investigated thus far.

## Introduction and background

The first electromagnetic experiment on conscious humans was performed by Merton and Morton in 1980s in which they electrically stimulated the motor cortex through the scalp using transcranial electrical stimulation (TES) [1]. An electric field was generated by placing the electrodes over the head that were connected to a high-capacity condenser and charged up to 2,000 volts. Unfortunately, through TES, only a fraction of current passed through the scalp while the rest spread between the electrodes that resulted in a painful contraction of the scalp muscles and caused significant pain and discomfort. A few years later, Barker and colleagues proposed a method of transcranial magnetic stimulation (TMS) that could replace TES. TMS used a magnetic field via a wire coil [2]. It was based on the principles of magnetic field induction as described by Faraday’s law. In this technique, the investigators placed a coil near the scalp. A brief and rapidly changing high-intensity electrical current was then passed through it, which generated a powerful magnetic field capable of producing an electric current across the brain tissue. The induced current then depolarized nearby neural networks located beneath the coil and produced neurophysiological and behavioral effects [3].

Recently, TMS has recently become popular among the non-invasive brain stimulation techniques. These techniques aim to target specific cortical areas and induce patterns of energy to modify neural patterns of activity in the brain. However, it is noted that TMS can produce sufficient electric current to depolarize superficial axons and networks in the cortex; the strength of the current reaching the subcortical areas, including the basal ganglia and thalamus, is weak and cannot be stimulated [4]. This review introduces the concept of TMS, its mechanism, and a general view on the extent of its clinical applications in the past few years.

## Review

Electrophysiology of transcranial magnetic stimulation

**Figure 1:**
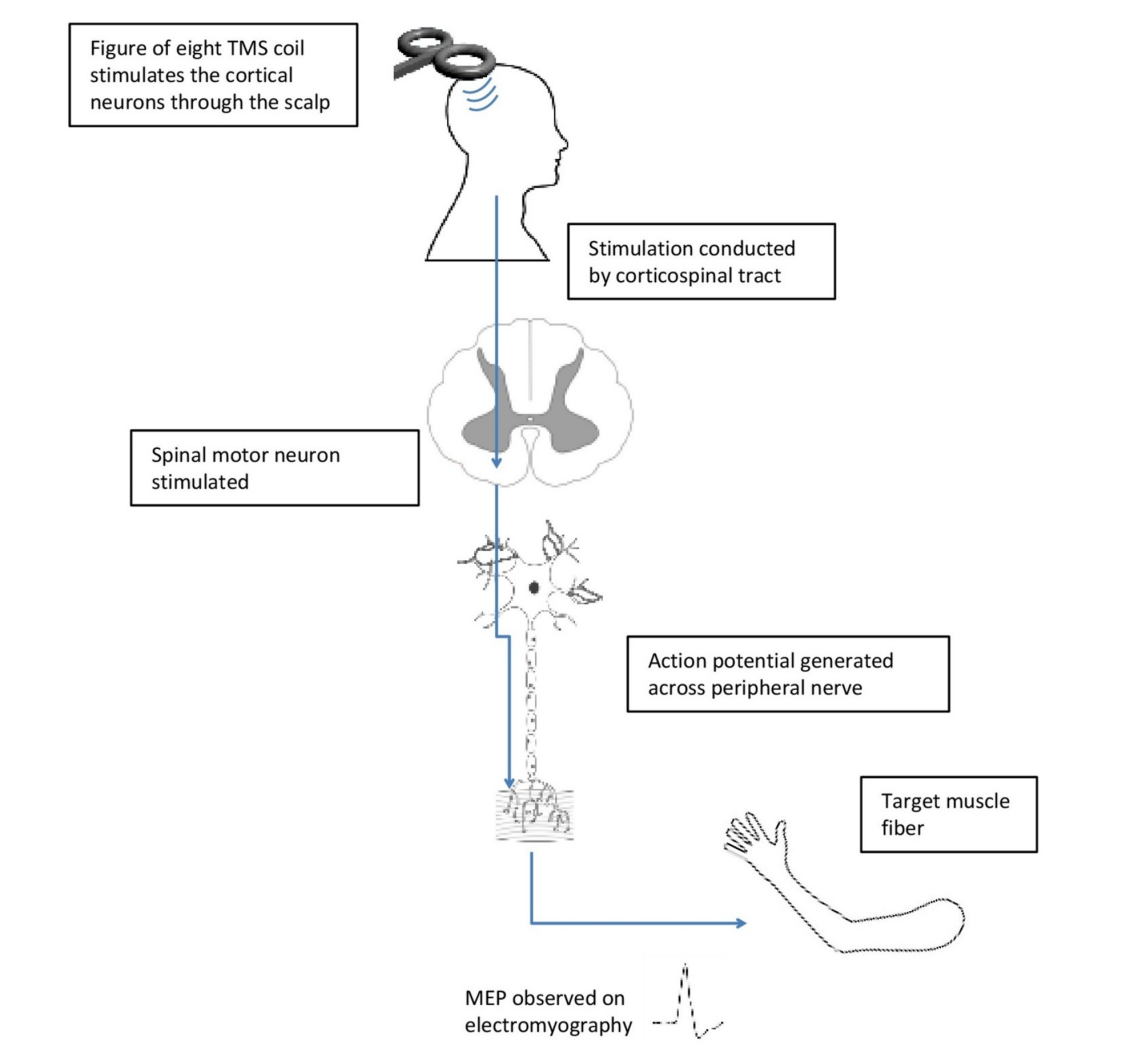
A diagrammatic representation of TMS stimulation of the motor pathway leading to muscle contraction MEP: motor-evoked potential; TMS: transcranial magnetic stimulation

In the 1990s, Tofts demonstrated that TMS induced circular electrical currents in the central nervous system through rapid changes in magnetic fields and preferentially stimulated the neurons that fall in a plane parallel to the coil [5]. For instance, when TMS is applied over the part of the scalp overlying the motor cortex, it induces discharges that run down the pyramidal tract and stimulates the spinal motoneurons (corticospinal tract). This results in a motor-evoked potential  (MEP) that can stimulate the muscle to move and can also be picked up by electromyography. The amplitude of the MEP and motor threshold (MT), which is the minimum TMS intensity required to generate a motor evoked potential of 50 mV, are two parameters that are used to estimate the excitability of the corticospinal tract (Figure 1) [6].

Motor units are recruited from the smallest to the largest, according to the Henneman size principle [7]. In 1987, a study demonstrated that a motor unit recruited during minimal voluntary contraction was also that recruited by TMS of the motor cortex; it was also noted that the motor unit was recruited in the same order like that with voluntary contraction [8].

To determine motor cortical and corticospinal excitability, TMS uses MT and MEP, which are based on various physiological mechanisms. MT depends on the excitability of cortico-cortical axons and excitatory pathways to corticospinal neurons. Agents that block voltage-gated sodium channels and those that act on ionotropic non-N-methyl D-aspartate (non-NMDA) glutamate receptors, such as ketamine, affect the MT [9]. Neurotransmitters, such as gamma-aminobutyric acid (GABA), acetylcholine, dopamine, serotonin, or norepinephrine, do not affect MT. MEP can also be blocked by agents that block sodium channels, such as volatile anesthetics [10]. The sodium channel inactivation leads to decreased action potential firing that, in turn, reduces calcium entry at the presynaptic terminal and finally synaptic transmission resulting in reduced MEP [11].

MEP amplitude was also found to be affected by the modulators of inhibitory and excitatory transmission in neuronal networks. The neurotransmitter modulators for GABA-A receptors depressed MEP while dopamine agonists and norepinephrine agonists raised the MEP. Hence, there is a difference between the physiologies of MT and MEP [12].

In 1990, Burke et al. compared descending volleys evoked by TES and TMS in the corticospinal tracts of animal models [13]. The corticospinal volleys evoked by TES recruited motor units in a similar pattern as those evoked in animals by electrical stimulation of the motor cortex that concluded the fact that TES stimulates cortical neurons in a plane vertical to the surface of the brain [14], whereas the recruitment pattern of TMS differed from that of TES, as demonstrated in the Tofts model. TMS preferentially activated cortical interneurons transmitting excitatory inputs to the pyramidal neurons.

The electrical field generated in the brain by TMS depends on many physical and biological parameters, such as the frequency, intensity, and pattern of stimulation, the magnetic pulse waveform, orientation of the current lines evoked in the brain, orientation and shape of the coil, and excitable neural elements. TMS can deliver a monophasic pulse or biphasic pulses as discussed later in the article [4].

Apart from affecting the electrical activity of the brain, it is postulated that TMS might also affect the blood-brain barrier permeability [15], neuronal metabolism [16], and protein signaling and transcription [17].

Moreover, TMS might affect the brain via non-electromagnetic pathways as well (for example, the stimuli from sounds from the machine, pressure stimuli from overlying coils placed on the scalp, or even secondary afferent effects from peripheral nerve activation in the vicinity) [18]. This will require further research to determine its accuracy and effectivity and is beyond the scope of this article.

Types of TMS pulses

There are three main types of pulses (Table 1).

**Table 1:** Types of TMS pulses CS: conditioning stimulus; ISI: inter stimulus interval; ms: milliseconds; sec: seconds; TMS: transcranial magnetic stimulation; TS: test stimulus

Types of pulse	Definition	Effect
Single pulse	Discharge of single pulses separated by a time interval of at least 4 sec to 8 sec, resulting in an individual effect constitutes single pulse-TMS.	The induced effects can be quantified in the form of 1) motor evoked activity in primary motor areas; 2) evoked visual activity and visual precepts, such as phosphenes; 3) short-acting disturbances in cognitive tasks, such as changes in performance.
Double pulse	Also known as paired pulse-TMS, it consists of two pulses, i.e., discharge of a CS followed by a TS that are separated by an ISI.	By using subthreshold CS and suprathreshold TS and using varying ISI from short (< 5 ms), intermediate (7 - 15 ms), and long (50 - 200 ms), the effect can be modulated from intracortical facilitation to inhibition. They can also be used to determine the presence or absence of connectivity and estimation of conduction time between two distinct cerebral sites
Repetitive pulse	Repetitive pulse-TMS consist of delivering any combination of more than two pulses with in-between time interval of 2 sec or less to generate different effect than that produced from the isolated pulse. It involves delivery of short bursts or trains of 3 - 4 pulses at high frequency and of long periods of stimulation at a fixed frequency with or without interruption by stimulation free intervals in between charges.	The effects can range from 1) online TMS effect: This results from direct and measurable interference with patterns of ongoing neuronal discharge at the time of stimulation; 2) offline TMS effect: This constitutes the lasting impact on the cerebral process that results from a previously administered pattern of repetitive stimulation.

Coils and their characteristics

*Types of Coils*            

Depending upon their difference in size, dimensions, and electric field characteristics, various types of TMS coils have been developed and range from simple geometry circular coils to the more complex figure-8 coil, double cone coil, the Hesed coil, the slinky coil, cloverleaf coil, the -D differential coil, C-core coil, air-cooled coil, and the circular crown coil.

General Characteristics of Coils

During selection of TMS coils, the two major areas of interest related to electric field characteristics are the (a) focality and (b) depth of brain stimulation. Coils with larger dimensions have a deeper penetration due to slower electric field attenuation but with less focality, although smaller coils have better focality over larger coils in terms of activated brain volume. However, this advantage diminishes with increasing depth of the target region, such as the cortical lower limb region.

Electric Field Characteristics of the Individual Coil

Circular coil: This non-focal, ring-shaped coil induces an electric field that stimulates a broader region of the brain present beneath the coil perimeter [19].

Figure-8 coil: This coil consists of a pair of adjacent circular loops with current flowing in the opposite direction and provides relatively focused electric stimulation under the coil at the point where the two rings intersect each other [19].

Cloverleaf coil: This coil consists of four coils of nearly circular windings and better stimulates long fibers as compared to figure-8 coils [20].

Slinky coil: This coil is composed of multiple circular or rectangular loop windings joined together at one edge while fanned out from another to form a half toroid. It can achieve larger field magnitude and better focality at the cortex near the coil center [19-20].

Three-dimensional (3-D) differential coil: This coil consists of a small figure-8 coil with a third loop present perpendicular to its center and surrounded by two additional loops to limit the area of stimulation. It has the advantage of more focal stimulation when compared to figure-8 and slinky coils [19].

Double cone coil: This coil consists of two large adjacent circular windings fixed at an angle to each other. Compared to the figure-8 coil, the stimulation produced does have a deeper penetration but a less focal electric field [20].

Hesed (H) coil: With more complex winding patterns and larger dimensions compared to the conventional TMS coils, the H coils can stimulate deeper brain regions more effectively because of their slower electric field decay at depth but at the expense of decreased focality. The high permeability ferromagnetic cores have been introduced in recent advances with the hope to improve the focality, penetration, and electric field efficiency of these coils [19-20]

Other coil designs: The C-core coil, circular crown coil, the large halo coil, and MRI gradient coil designs with larger dimensions than conventional and H coils have also been under investigation for deeper TMS with the expectation of slower electric field decay at the expense of reduced focality [19].

Clinical application of TMS in psychiatric disorders

Obsessive-compulsive Disorder (OCD)

OCD affects about 1% to 2% of the Western population. The obsessions are characterized by recurring thoughts that are difficult to prevent or control and could lead to irresistible and recurring behaviors, also known as the compulsions. The compulsions are actions that are taken to negate or resolve the disruptive, anxiety-provoking, and disturbing thoughts. A familiar example is of an individual who fears “contamination” and feels compelled to cleanse their hands with disinfectants repeatedly. The unwanted obsessive thoughts frequently disrupt personal relationships, work, sleep, and almost all other aspects of daily life.

Up to 60% of patients with OCD do not respond to first-line treatment. In a meta-analysis of 20 single- or double-blind, randomized studies, researchers in China examined the efficacy of repetitive transcranial magnetic stimulation (rTMS) in 791 patients with OCD [21]. Overall, rTMS showed a large effect size on OCD symptoms compared with the sham condition immediately after treatment lasting for two to 12 weeks. High- (HF) and low-frequency (LF) rTMS had approximately equal efficacy, as did the treatment that targeted the supplementary motor area and the left, right, and bilateral dorsolateral prefrontal cortex. Larger effects were associated with not having comorbid major depression, lack of resistance to other therapies, and receiving 100% resting motor threshold intensity rTMS. Tilted coils produced larger effects than sham coils, indicating that sham coils have a larger placebo effect and possibly more effective blinding [21]. However, these therapeutic effects are short-term, and further meta-analyses are yet to show long-term therapeutic effects.

Major Depression (MD)

Major depression is diagnosed in the presence of five or more of the following symptoms for at least two consecutive weeks: depressed mood, loss of interest or pleasure in most or all activities, insomnia or hypersomnia, significant weight loss or weight gain or decrease or increase in appetite, psychomotor retardation or agitation, fatigue or low energy, decreased ability to concentrate or make decisions, thoughts of worthlessness, excessive or inappropriate guilt, and thoughts of death or suicidal ideation or a suicide attempt [21].

Many patients with unipolar MD do not respond to standard treatment with pharmacotherapy and psychotherapy [22]. Although electroconvulsive therapy (ECT) is more efficacious than repetitive TMS, patients may prefer repetitive TMS because it is better tolerated, and unlike ECT, it does not require general anesthesia and induction of seizures. 

In a study conducted by Eranti et al. in 2007 to compare the efficacy of ECT with rTMS, 46 patients with major depression referred for ECT were randomly assigned to either a 15-day course of rTMS of the left dorsolateral prefrontal cortex (N = 24) or to a standard course of ECT (N = 22) [23]. Results were calculated based on the score on the 17-item Hamilton Depression Rating Scale (HAM-D) and the percentage of patients with remissions (Hamilton score of ≤ 8) at the end of treatment and six months follow-up, which were the primary outcomes of the study. Secondary outcomes included mood self-ratings on the Beck Depression Inventory-II and visual analog mood scales, Brief Psychiatric Rating Scale (BPRS) score, self-reported and observer-rated cognitive changes. HAM-D scores were calculated, also, at the end of the treatment and the six-month follow-up. It was found that the HAM-D scores at the end of treatment were significantly lower for ECT, with 13 patients (59.1%) achieving remission in the ECT group, while there were four (16.7%) in the rTMS group. However, at the six-month follow-up, the HAM-D scores did not differ between groups that were either treated with ECT or rTMS. Beck scale, visual analog mood scale, and BPRS scores, however, were lower for ECT at the end of treatment and remained lower after six months. Self- and observer-rated cognitive measures were similar in the two groups. Hence, rTMS was not as effective as ECT, and ECT was substantially more useful for the short-term treatment of depression but nearly equal in the long-term [23].

Meta-analyses have shown that HF-rTMS has antidepressant properties when compared with sham rTMS. Data from 29 RCTs were included, totaling 1,371 subjects with MD. Following approximately 13 sessions, 29.3% and 18.6% of subjects who received HF-rTMS were classified as responders and remitters, respectively. The results were compared with 10.4% and 5% of those who received sham rTMS (pooled odds ratio (OR): 3.3, p < 0.0001, with numbers needed to treat (NNT) of 6 and 8, respectively). Furthermore, it was found that HF-rTMS was equally effective as an augmentation strategy or as monotherapy for MD and when used among patients with primary unipolar MD or mixed with unipolar and bipolar MD [24].

Bipolar Disorder

Bipolar disorder is a mood disorder that is characterized by episodes of mania, hypomania, and major depression [25].  The subtypes of bipolar disorder include bipolar type I and type II. Patients with bipolar I disorder experience manic episodes and nearly always experience major depressive and hypomanic episodes. Bipolar II disorder is marked by at least one hypomanic episode, at least one major depressive episode, and the absence of manic episodes. Additional information about the diagnosis of bipolar disorder is discussed separately.

Many patients with acute bipolar major depression or mania do not respond to pharmacotherapy. Also, maintenance treatment with medications and psychotherapy often fails to prevent recurrent mood episodes. Patients unresponsive to standard treatment may be candidates for neuromodulation therapies [22].

In 1998, a study was done in which 16 patients completed a 14-day double-blind, controlled trial of right versus left prefrontal transcranial magnetic stimulation at 20 Hz (two-second duration per train, 20 trains per day for 10 treatment days) [26]. Mania was evaluated with the Mania Scale (MS), the Brief Psychiatric Rating Scale (BPRS), and the Clinical Global Impression (CGI). For total BPRS, two-way repeated measures analysis of variance (ANOVA) with covariance for baseline showed a significant effect of time (F = 3.9, df = 2, 28, p = 0.03) and a significant interaction of time and side of TMS (F = 3.4, df = 1.2, 17.2, p < 0.08, Greenhouse-Geiser-corrected). For the Mania Scale, two-way repeated measures ANOVA with covariance for baseline showed a highly significant effect of time (F = 16.0, df = 2, 28, p < 0.0001) and a significant interaction of time and side of TMS (F = 5.2, df = 1.4, 19.2, p = 0.04, Greenhouse-Geisser-corrected). For the BPRS mania factor, which includes the items excitement, grandiosity, hostility, tension, and uncooperativeness, two-way repeated measures ANOVA with covariance for baseline showed a significant effect of time (p = 0.02) and a significant interaction of side of TMS and time (F = 3.4, df = 1.7, 23.2, p = 0.05, Greenhouse-Geisser-corrected). For the CGI, two-way repeated measures ANOVA with covariance for baseline showed a highly significant effect of time (F = 14.6, df = 2, 28, p < 0.0001) and a significant interaction of time and side of TMS (F = 3.9, df = 1.5, 28, p = 0.06, Greenhouse-Geisser corrected) [26].

In 2014, a group of European experts was commissioned to establish guidelines on the therapeutic use of rTMS through the collection of data published up until March 2014. The data included the use of rTMS in various neurological and psychiatric conditions, such as movement disorders, stroke, multiple sclerosis, epilepsy, depression, anxiety disorders, obsessive-compulsive disorder, schizophrenia, and addiction, among many others. Despite some unavoidable inhomogeneities, the collected evidence was sufficient to accept with level A (definite efficacy) the analgesic effect of HF-rTMS on the primary motor cortex (M1) and the antidepressant effect of HF-rTMS on the left dorsolateral prefrontal cortex (DLPFC). A Level B recommendation (probable efficacy) was proposed for the antidepressant effect of LF-rTMS of the right DLPFC, HF-rTMS of the left DLPFC for the negative symptoms of schizophrenia, and LF-rTMS of contralesional M1 in chronic motor stroke. However, more research is needed to figure out how to optimize rTMS protocols and techniques that would give it relevance in routine clinical practice. Also, professionals carrying out rTMS protocols should undergo rigorous training that would specialize them into proper handling of the instruments used and would maximize the chances of success. Under these conditions, the therapeutic use of rTMS should be able to develop in the coming years [27].

Schizophrenia

Schizophrenia is diagnosed on the basis of its characteristic symptoms (positive symptoms, i.e., delusions, hallucinations, disorganized speech, or behavior, and/or negative symptoms, i.e., diminished emotional expression and lack of motivation) coupled with social and/or occupational dysfunction for at least six months in the absence of another diagnosis that would explain for the presentation. Schizophrenia is considered to be the most debilitating of psychiatric illnesses, psychologically, socially, and financially. Although pharmacotherapy, especially antipsychotics, remains the mainstay in the acute treatment and maintenance of schizophrenia, an alternate treatment modality (such as TMS) is needed due to the limitations of antipsychotic agents.

Literature review in the last 15 years supports the fact that TMS is a safe and efficacious means of treating the positive and negative symptoms of schizophrenia. The most notable body of evidence supports the reduction of auditory hallucinations by targeting LF-TMS stimuli to Wernicke’s area in the left temporoparietal cortex [28]. The aforementioned conclusion was not based on the results of all the studies unanimously; some studies also showed negative results for the efficacy of TMS.

Almost a quarter of patients with schizophrenia present with resistant auditory verbal hallucinations (AVHs), a phenomenon that may relate to activation of brain areas underlying speech perception. In 2005, Poulet et al. conducted a study compromising of 10 right-handed schizophrenia patients with resistant AVH who received five days of active rTMS and five days of sham rTMS (2,000 stimulations per day at 90% of motor threshold) over the left temporoparietal cortex in a double-blind crossover design. AVHs were robustly improved (56%) by five days active rTMS, whereas no variation was observed after sham treatments. Seven patients were responders to active treatment, five of whom maintained improvement for at least two months [29].

In 2007, Prikyl et al. conducted a study which concluded that augmentation of rTMS enabled patients to experience a significant decrease in the severity of the negative symptoms. During the real rTMS treatment, a statistically significant reduction of negative symptoms was found - approximately a 29% reduction in the parasympathetic autonomic nervous system (PANS)-negative symptom subscale and a 50% reduction in the sympathetic autonomic nervous system (SANS) [30].

rTMS is noted to be greater if it is used at a frequency of stimulation of 10 Hz, 110% motor threshold, and stimulates the left DLPFC. Moreover, positive results are observed in illnesses that have a longer duration of treatment (at least three consecutive weeks) and a shorter duration of disease [31]. Although TMS showed promising results in a few studies, large-scale studies are required to conclude its efficacy in patients with schizophrenia.

Autism Spectrum Disorders

Autism spectrum disorder (ASD) is a biologically based neuro-developmental disorder which is characterized by deficits in social communication and interaction and restricted, repetitive patterns of behavior, interests, and activities [25]. ASD is diagnosed clinically, based on the presence of key behavioral symptoms, but the underlying pathophysiology in the brain behind these symptoms is unknown. The treatment is mainly supportive with a focus on early intensive behavioral interventions [32].

Single pulse TMS: In ASD, single pulse TMS has been used to probe baseline levels of corticospinal excitability and modulation of corticospinal excitability in response to visually presented stimuli. Six independent studies have shown no difference in either motor threshold (the lowest intensity of stimulation required to induce an MEP) or the size of MEP in response to a suprathreshold pulse of TMS between individuals with ASD and neurotypical individuals. The studies suggest that baseline M1 excitability is not affected in ASD [33].

LF-rTMS: In 2014, Sokhadze et al. studied whether 1 Hz rTMS improves electrocortical functional measures of information processing and behavioral responses in autism [34]. Post-TMS evaluations showed decreased irritability and hyperactivity on the Aberrant Behavior Checklist (ABC) and decreased stereotypic behaviors on the Repetitive Behavior Scale (RBS-R). Following the rTMS course, they found decreased amplitude and prolonged latency in the frontal and frontocentral N100, N200, and P300 (P3a) and event-related potentials (ERPs), which are small voltages generated in the brain in response to specific events or stimuli; N100 - N300 are the negative deflections after presentation of stimulus, whereas P100 and P300 are positive wave deflections after the stimulus [35]. TMS resulted in an increase of P2d (P2a to targets minus P2a to non-targets) amplitude. ERP changes along with increased centroparietal P100 and P300 (P3b) to targets were indicative of the more efficient processing of information post-TMS treatment. Enhanced information processing was also noted in a lower error rate [34].

HF-rTMS: The only study in the published literature that included participants with intellectual disability was conducted by Paerai et al. in 2013 where high-frequency 8 Hz rTMS was applied to the left premotor cortex in children with ASD with intellectual instability [33]. The report, based on four studies with children with low-functioning autism, aimed at evaluating the effects of rTMS delivered on the left and right premotor cortices (PrMC) on eye-hand integration tasks, defining the longlasting effects of HF-rTMS. The study investigated the efficacy of high-frequency rTMS by comparing three kinds of treatments: HF-rTMS, a traditional eye-hand integration training, and both treatments combined. Results showed an increase in eye-hand integration performances only after HF-rTMS was delivered on the left PrMC (mean increased performances = +4.11; standard deviation (SD) = 2.61; in the remaining conditions, mean increased performances ranged from −0.11 to +1). It also showed that effects of HF-rTMS in increasing eye-hand integration performances turned out to be rather persisting up to one hour after the end of the left-PrMC stimulations [33]. Based on these preliminary findings, further evaluations on the usefulness of HF-rTMS in the rehabilitation of children with autism are strongly recommended.        

Conversion Disorder

Conversion disorder is described as neurological signs and symptoms, such as movements, seizures, or sensory symptoms, without an underlying neurological or medical cause. Symptoms usually follow a psychosocial or traumatic life experience. Subconscious psychological factors are judged to be associated with the symptoms because of a temporal relation between a psychosocial stressor and initiation or exacerbation of a symptom [25].

A variety of treatment options have been tried to treat conversion disorder. TMS as a therapy for the reversal of conversion disorder symptoms is now being applied to various patients with a promising future. For instance, in one experiment, four patients were treated over a period of five to 12 weeks with rTMS applied to the contralateral motor cortex [36]. In one patient, motor function was completely restored; two patients experienced a marked improvement correlating with rTMS treatment.

Research in conversion disorder has been quite neglected. It is partly attributed to the fact that not many such patients report to the doctors. A larger sample size study is needed to understand this disorder further and experiment with various therapies to treat successfully. rTMS, in particular, is promising in that it has been successful as a treatment modality in other psychiatric illnesses, like depression.

Eating Disorders

The use of transcranial magnetic stimulation can aid in decreasing some of the behaviors and symptoms that are seen with bulimia, but for a short period. Various brain stimulation techniques have been studied for their effectiveness with eating disorders, and transcranial magnetic stimulation has had success.

Bulimia nervosa is a psychiatric disorder which is characterized by cyclical binging overeating episodes characterized by a subjective loss of control, followed by extreme or inappropriate compensatory behaviors to expunge calories taken in during the binge episodes. These patients use laxatives, exhibit self-induced purging behavior, or exercise excessively. Anorexia may also include binging and purging, but its hallmark feature is the active maintenance of dangerously low body weight for age, sex, and developmental trajectory. Anorexia is coupled with a strong drive for thinness and intense fear of weight gain. Both bulimia and anorexia can include significant body image distortions [25].

Currently, family-based therapy for anorexia and cognitive behavioral therapy for bulimia is used along with anti-depressants. However, relapse rates are high in anorexia and bulimia. Because of the serious impact of eating disorders and incomplete efficacy of existing interventions, new treatment modalities are of significant interest and transcranial magnetic stimulation is no exception.

Studies show that rTMS methods yield a statistically significant effect on eating disorders, though the source of such methodological variability remains uncertain (i.e., site of stimulation, stimulation technique, or other unknown factors) [37]. Further findings from a meta-analysis revealed that TMS techniques did not yield a significant effect in a single-session format for actual food consumption [38]. However, the relatively limited number of studies and significant methodological heterogeneity in an assessment of observed dietary behavior makes the null effect somewhat challenging to interpret. Trials have shown a reduction in short-term binging episodes among bulimic patients and no difference in long-term binging. Concerning anorexia, the benefits of TMS have been significantly more unclear. However, in clinical trials, among the symptoms affected by TMS, anxiety and stress appeared to be most improved [37]. In summary, it is difficult to recommend excitatory rTMS as a definite treatment for bulimia or anorexia.

Clinical applications of TMS in neurological disorders

Drug-resistant Epilepsy

Epilepsy is marked by altered cortical excitability that affects an estimated one in 26 individuals over their lifetime. Only two-thirds of patients with epilepsy respond to antiepileptic drugs (AEDs). The rest of the one-third of patients has drug-resistant epilepsy. These patients are at an increased risk of morbidity. Low-frequency TMS plays a critical role in the management of drug-resistant epilepsy, especially for patients with the type of epilepsy not suitable for surgical ablation. A combination of various frequency, strength, and type of coils are used to achieve varying degrees of transcranial magnetic stimulation.

A meta-analysis of 12 studies investigating the use of low-frequency (≤ 1 Hz) rTMS for the treatment of drug-resistant epilepsy, found a significant reduction in overall seizure frequency over an average follow-up period of six weeks. Also, rTMS was found to be more effective in patients with a mean age of < 21 years. In studies without IPD, targeted stimulation was associated with the strongest treatment effect. Similarly, in an analysis of individual participant data (IPD), a univariate analysis of the coil type revealed that the use of a figure-8 coil was associated with greater treatment response and extratemporal seizure focus predicts worse treatment outcomes [39]. The meta-analysis also identified a 30% reduction in seizure frequency following LF-rTMS for the treatment of refractory epilepsy. This effect is consistent with a previous meta-analysis by Hsu and colleagues that identified a 34% reduction in seizure frequency following rTMS treatment [40].

More research with a large sample, using different frequencies of pulses, is needed to prove if TMS could be a therapeutic option for epilepsy. It should be noted that in the studies quoted, very few participants achieved full seizure remission; however, the vast majority of participants experienced a therapeutic reduction in seizure frequency.

Multiple Sclerosis

Multiple sclerosis (MS) is an autoimmune, chronic central nervous system disease of unknown etiology. It presents as an ongoing demyelinating, inflammatory, and degenerative process, affecting both grey and white matters of the brain and the spinal cord. It results in the accumulation over the years of disabling motor and cognitive handicaps affecting the personal, professional, and social quality of life.

Studies have revealed that repeated stimulation of a single neuron at low-frequency produces longlasting inhibition of cell-cell communications; conversely, repeated high-frequency stimulation can improve cell-cell communication [39]. Trains of rTMS pulses can induce modification of activity in the targeted brain region, which can last for minutes or even hours. Various disease-modifying drugs are used for the treatment of MS. TMS has no interaction with such drugs and can be used as an adjunct to manage motor and sensory symptoms of MS, such as pain, fatigue, and spasticity. Based on the recent guidelines, there are still no recommendations for the therapeutic use of repetitive TMS in MS patients. Burhan et al. reported better gait performance as assessed by an electronic walkway system after the application of rTMS in a patient with a four-year history of relapsing-remitting MS presenting with cognitive and gait abnormalities. They showed a shorter ambulation time (time elapsed between the first contact of the first and the last footfalls, measured in seconds), faster gait velocity in response to three rTMS daily sessions, and an increase of cadence after one and three sessions [40].

Based on the findings, it cannot be concluded whether TMS could be considered as a therapeutic measure for MS. These results were obtained through the application of various rTMS protocols over the motor cortex for improving spasticity and ambulation in MS patients, and these results are needed to be confirmed by conducting studies on a bigger sample size.

Movement Disorders

Recent studies suggest that repeated transcranial magnetic stimulation (TMS) improves functional movement disorders (FMDs), such as dystonia, tremor, myoclonus, and Parkinsonism, but the underlying mechanisms are unclear. Therapeutic efficacy of TMS in patients with FMDs is mainly due to a cognitive-behavioral effect rather than neuromodulation.

The suprathreshold intensity of TMS might thus be an essential prerequisite for efficacy. During suprathreshold magnetic stimulation sessions, patients experience the unexpected stimulation-induced movement of their affected limbs. This may make the patient realize that his or her motor system is working properly and thereby allow the brain to “relearn” or “reprogram” a normal pattern of movement [41]. It is this relearning of the normal pattern of movement that contributes to the longlasting therapeutic effect of TMS.

Parkinson’s disease (PD) has wide-ranging clinical features, and rTMS therapy has been tried for many aspects of PD. The underlying mechanism, however, remains unclear; however, several possibilities are proposed, such as endogenous dopamine release or restoration of network activity. Motor symptoms are a cardinal feature of PD, for which evidence suggests the moderate efficacy of rTMS. HF-rTMS over the M1, including less focal stimulation (e.g., leg and bilateral hand M1 rTMS) or over the DLPFC, and LF-rTMS over the supplementary motor area (SMA) were most favorable. rTMS is reportedly also effective for levodopa-induced dyskinesia (LID), which is caused by long-term administration of levodopa among PD patients. rTMS has also been tried for non-pharmacological treatment of non-motor symptoms of PD, including depression. A “weak recommendation” in favor of HF-rTMS of the left DLPFC is given for the treatment of depressive symptoms associated with PD.

These are examples of the growing application of rTMS therapy for PD for symptoms other than the classic motor symptoms. As such, rTMS has a potential to become an important adjunctive treatment for PD. Well-designed large clinical trials are still needed to establish its utility in the clinical settings [42].

Migraine

A migraine is a type of chronic headache related to cortical excitability. TMS has been shown to activate or suppress the cortex excitability. Because there are limited drugs that can improve the quality of life for people with a migraine, TMS is a promising therapy. It can facilitate or inhibit the electrical activity of the cerebral cortex. Some existing randomized clinical trials (RCTs) reveal that TMS can relieve a headache.

Nonetheless, there are a few meta-analyses discussing the effect of TMS on migraine. A meta-analysis reported some studies in which HF-rTMS was effective and well tolerated for migraine prophylaxis [43]. In another study, no statistically significant difference between LF-rTMS with sham stimulation was found.

When the effect of TMS on chronic migraine was evaluated, it was concluded that there was no statistically significant difference in effect between the active TMS group and sham TMS group. In light of this, the hypothesis that was put forward suggested that TMS can change the excitability of cortex. However, it needs more time to do this. Unfortunately, due to the small sample size, this conclusion was not definite. More well-designed RCTs are needed to confirm this conclusion.

The reasons for variability of TMS results in various patients is not only the dose but also the side, location of stimulation, type of coil, and the number of sessions. Nevertheless, at present, there is no common standard of TMS for migraine. For a chronic migraine, TMS is even more ineffective as chronic pathologic changes in excitability of cortex in a chronic migraine would need a longer duration of TMS to show any effect [43].

Fibromyalgia

Fibromyalgia is the presence of widespread musculoskeletal pain that cannot be attributed to any cause. Evidence from recent studies indicates that the pain in fibromyalgia springs from CNS augmentation of pain centers in the brain. Gracely and colleagues found that approximately 50% lower stimulus intensity was needed to evoke a pain response in patients with fibromyalgia as compared to healthy controls (P < 0.001) [44]. In the background of this mechanism, TMS therapy targeted on the motor cortex has been successfully employed for the treatment of these patients, probably by modifying these pain centers in the brain. A study conducted in 2001 showed that unilateral rTMS of the motor cortex induces a long-lasting decrease in the widespread chronic pain experienced in fibromyalgia [45]. Another study conducted in 2010 suggested long-term improvement related to the quality of life (including fatigue, morning tiredness, general activity, walking, and sleep), which were reported to be directly correlating with changes in intracortical inhibition [46]. However, a recent meta-analysis on the efficacy of rTMS for fibromyalgia showed mixed outcomes. rTMS improved quality of life among patients with fibromyalgia with a moderate effect size (Pooled standardized mean difference (SMD) = −0.472 95%CI = −0.80 to −0.14); it also showed a trend toward reducing pain intensity (SMD = −0.64 95% confidence interval (CI) = −0.31 to 0.017) but did not change depressive symptoms [47].

In view of this, further investigations and trials based on using different frequencies of rTMS or combining it with other treatment modalities (like serotonin, which is found to be low in the serum of patients with fibromyalgia) to treat the disorder are needed to establish a consequent and firm efficacy or non-efficacy of rTMS to treat pain in fibromyalgia.

Tinnitus

Tinnitus, also known as phantom auditory perception, describes the conscious perception of an acoustic sensation in the absence of a corresponding external stimulus [48]. The American Academy of Otolaryngology guidelines for the treatment of tinnitus includes stress reduction, cognitive therapy, masking, and sleep improvement [49]. In regard to the neuronal and central auditory involvement in the pathophysiology of tinnitus, TMS therapy is emerging as a new treatment modality with promising results. A study showed that rTMS did produce changes in the auditory cortex. Moreover, it was found that only some patient characteristics, such as patients with shorter duration of tinnitus, normal hearing, and those with little or no sleep disturbance, showed a significant reduction in tinnitus after TMS [50].

## Conclusions

Research on TMS as a treatment option for various neurological and psychiatric illnesses has increased in the recent years. The aim of this review was to shed light on the basics of TMS and to summarize its use in various diseases. Although TMS showed some efficacy in treating symptoms of various neurological and psychiatric illnesses, further investigation is required with large sample sizes and comparison with standard treatments. Based on these studies, TMS could also serve as a good diagnostic and research tool in the field of neurosciences.
